# Supplementary material for: Haploflow: strain-resolved de novo assembly of viral genomes
Source: Genome Biol. 2021 Jul 19;22:212. doi: 10.1186/s13059-021-02426-8 (PMC8287296; doi:10.1186/s13059-021-02426-8)
Supplement: Supplementary file 1 — Additional File 1:. Fig. S1-S4 and Tables S1-S8 [file 13059_2021_2426_MOESM1_ESM.pdf]

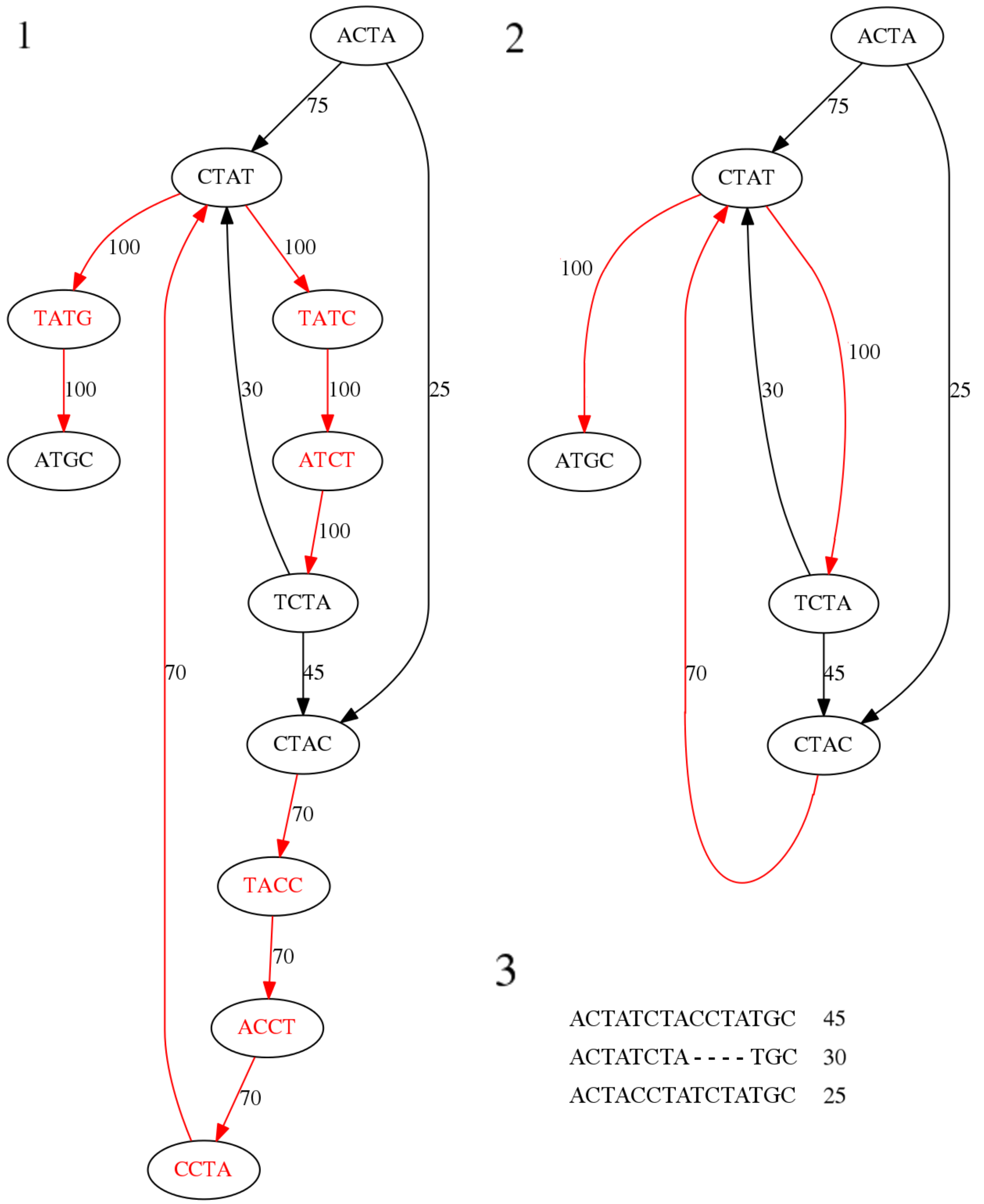

Figure S1: The deBruijn graph (1) and its corresponding Unitig graph (2) for three related sequences and their coverage (3). The red *k*-mers and edges between them are part of linear paths and are replaced by a single red edge in the unitig graph. The edges are labelled with the “capacity”, the sum of the coverages of the sequences going over them, in the deBruijn graph and the average capacity of all smoothed edges in the Unitig graph - which in this case

is the same as the original capacity. Some of the edges represent one (capacities 25, 30, 45), some two (capacity 70 = 45 + 25) and some all (capacity 100 = 45 + 30 + 25) of the sequences.

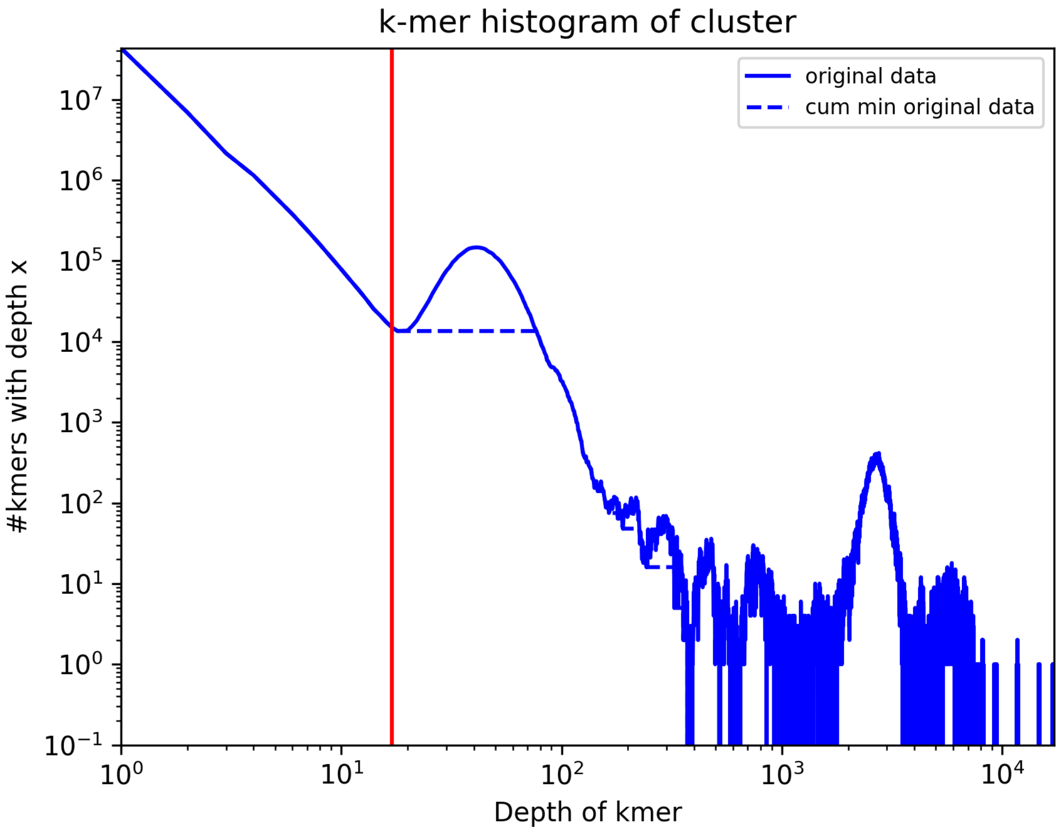

Figure S2: log-log kmer coverage histogram for a sequence sample of HCMV strain TB40E and *E. coli.* This shows the kmer coverage (or counts) on the x-axis versus the number of kmers with that coverage on the y-axis. Original values are shown with the solid line, the cumulative minimum of the number of k-mers of a certain depth with the dashed line. k-mers with a depth less than the depth of the first k-mer for which the cumulative minimum (cum min) is less than the original value are regarded as probable erroneous k-mers (red line). For example, for a mixture of *Escherichia coli* (5,129,110 bp) and HCMV (234,127 bp), with a length ratio of 22:1, distinct peaks occur at coverages of ~45 and ~2500. The first peak has 10,000 distinct kmers and the second one 400, indicating that the first genome might be around 25x as large as the second one.

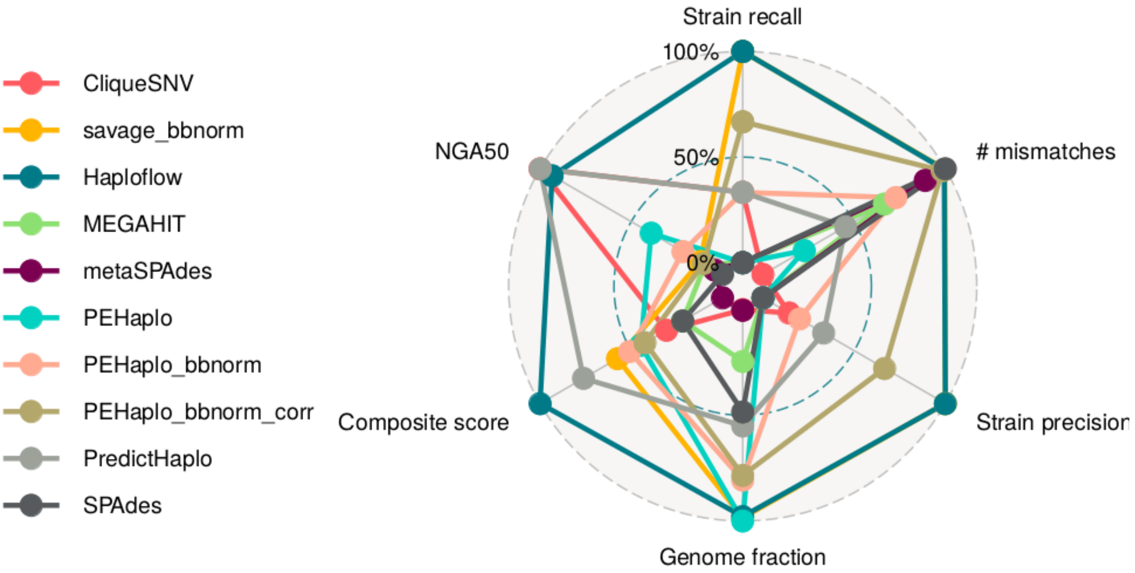

Figure S3: Radar plot of relative performance for Haploflow and nine other methods for the HIV-3 *in silico* data set. Best performance is at 100% and Haploflow, in dark blue, ranks first in Strain recall, Strain precision and composite score.

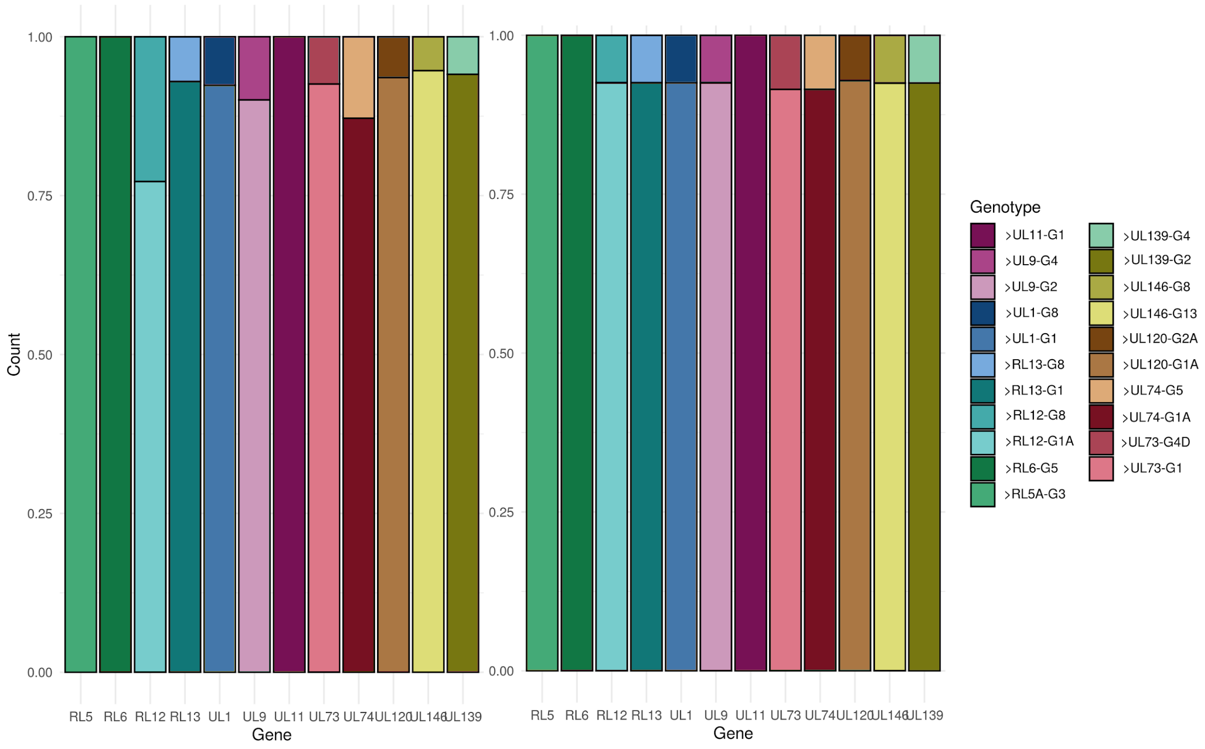

Figure S4: Genes with different genotypes and their coverage distribution in the reads (left) and in the contigs (right). VATK was run on the reads and the contigs. For the contigs the bar height was set based on the coverage value Haploflow reports. VATK found the exact same genotypes in the reads and the Haploflow contigs.

Table S1: SNPs and short indels detected by Haploflow and Lofreq for all 17 samples of SARS-CoV-2 in at least one sample. Lofreq was run with default parameters and SNPs were filtered by a score of >1000 and abundance >5%. “Rare” indicates thatLofreq predicted this variant at less than 5%, homopolymeric means that this site is located within or in the direct vicinity of a 4bp or longer homopolymer.

Position	Original base	Detected base	Detected by	Notes
518-520	ATG	---	Haploflow	
686-694	AAGTCATTT	---------	Haploflow	
1440	G	A	Haploflow, Lofreq	
2891	G	A	Haploflow	
4802	G	A	Lofreq	homopolymeric
7717	T	A	Haploflow, Lofreq	
10507	C	T	Haploflow, Lofreq	
11335	G	T	Haploflow, Lofreq	
11454	C	T	Haploflow, Lofreq	
11467	G	T	Haploflow, Lofreq	
11514	C	T	Haploflow, Lofreq	
11897	C	A	Haploflow, Lofreq	
12071	G	A	Haploflow	rare
13115	C	T	Haploflow, Lofreq	
15139	A	C	Haploflow, Lofreq	
15157	C	A	Lofreq	Strand bias, called by Haploflow then filtered
15168	G	A	Haploflow, Lofreq	homopolymeric
16954	C	T	Haploflow, Lofreq	
18110	C	A	Haploflow	rare
17373	C	T	Haploflow, Lofreq	
19182	A	G	Haploflow, Lofreq	

19610	C	T	Haploflow, Lofreq	
20298-20300	ATT	---	Haploflow	
21077	C	T	Lofreq	homopolymeric
21575	C	T	Haploflow	homopolymeric
22323	C	T	Haploflow, Lofreq	
25658	C	T	Haploflow, Lofreq	
29659	C	T	Lofreq	homopolymeric
29760	T	C	Lofreq	

Table S2: Number of SARS-Cov-2 genomes assembled by Haploflow from seven SARS-CoV-2 wastewater metagenome samples and GISAID IDs of identical genomes recovered from clinical isolates. Strains are listed in order of their estimated abundances for individual samples.

Sample	# strains Haploflow	GISAID matches to strains
Oakland 5/19	2	hCoV-19/USA/LA-SR0328/2020<br>hCoV-19/USA/CA-CSMC67/2020
Oakland 5/19 (2)	2	hCoV-19/Poland/PL_P31/2020<br>hCoV-19/Beijing/DT-BJ01/2020
Oakland 5/28	2	hCoV-19/USA/CA-CSMC25/2020<br>hCoV-19/USA/CA-CSMC67/2020
Oakland 6/09	1	hCoV-19/France/IDF-10064DR/2020
Oakland 6/30	2	hCoV-19/USA/WA-UW-11903/2020<br>hCoV-19/France/IDF-10064DR/2020
Oakland 6/30 (2)	2	hCoV-19/USA/CA-CSMC25/2020<br>hCoV-19/USA/LA-SR0328/2020
Marin 7/1	2	hCoV-19/USA/VA-DCLS-1271/2020<br>hCoV-19/USA/WA-UW-11903/2020

Table S3: Benchmark of Haploflow against five *de novo* assemblers and five reference-based assemblers (grey background) on the HIV-3 data set. For every metric, best performing methods (95-100% range of results) are indicated. *Strain recall*: fraction of correctly recovered high quality strain genomes (≤ 1 (≤ 5) mismatches per kb; more than 90% (80%) genome fraction); *Strain precision*: fraction of correctly recovered high quality strain genomes of all genome assemblies. Evaluation using metaQUAST results and derived strain assembly metrics with HIV reference genomes 89.6, HXB-2 and JR-SCF in "combined reference" mode. [a]Results for a 140 Mb subset of the 500 Mb data set generated with *BBnorm*. *runs that did not complete after ten days or failed. **as being an outlier, QuasiRecomb results were excluded from composite score and radar plot calculation.

	**Strain recall**	**Strain precision**	**Composite score**	**Genome fraction (%)**	**Number of contigs**	**Mismatches**	**Duplication ratio**	**NGA50**
Haploflow	**3/3**	**3/3**	**9.66**	**93.36**	**3**	**9**	**1.001**	**9083**
metaSPAdes	0/3	0/3	2.78	33.70	9	62	**1.005**	1246
SPAdes	0/3	0/3	4.28	63.09	20	**1**	**1.021**	864
MEGAHIT	0/3	0/3	4.28	48.47	12	257	**1.084**	1701
PEHaplo *l=40*	0/3	0/9	5.86	**94.63**	40	1208	3.106	4305
PEHaplo[a]	0(1)/3	1/5	6.28	82.80	20	363	1.683	2774
PEHaplo[a] *correct*	0(2)/3	2/3	5.7	81.45	17	**24**	**1.044**	1773
SAVAGE *de novo**	-	-	-	-	-	-	-	-
SAVAGE *ref**	-	-	-	-	-	-	-	-
SAVAGE[a] *ref*	*2(3)/3*	*3/3*	6.74	*93.72*	18	*5*	*1.057*	1932
PredictHaplo	1/3	1/3	8.02	67.12	3	631	1.497	*9658*
QuasiRecomb **	0/3	0/3,507	-	67.12	3506	1,011,054	1748.6	*9649*
QuasiRecomb *conservative* **	0/3	0/1,155	-	67.12	1154	411,576	575.67	*9654*
CliqueSNV	1/3	1/7	4.89	33.5	7	538	7.000	*9669*

GAEseq*	-	-	-	-	-	-	-	-
GAEseq[a]*	-	-	-	-	-	-	-	-

Table S4: Benchmark results for the HCMV data set. Shown are average values for the metaQUAST metrics over the six data sets and additional assembly metrics (see "performance evaluation"). For every metric, the best performing methods (95-100% range of results) are indicated. **Strain recall* includes correctly recovered genomes at two quality levels: more than 80(90)% genome fraction and less than 5(1) mismatches/kb. PEHaplo did not assemble one (TA-1-10) of the six mixtures.

	**Strain recall***	**Strain precision**	**Composite score**	**Genome fraction**	**Contigs**	**Mismatches per 100kb**	**Duplication ratio**	**NGA50**
Haploflow	**10(3)/12**	**10/14**	**9.34**	**83.87** ± **10.37%**	20.50 ± 7.20	166.26 ± 122.97	1.20 ± 0.15	**62,560.42 ± 35,233**
metaSPAdes	5(4)/12	5/12	8.18	58.57 ± 4.44%	17.42 ± 8.38	184.13 ± 337.14	**1.01** ± **0.01**	**60,008.25 ± 37,089**
SPAdes	6(4)/12	6/12	5.22	65.52 ± 3.94%	85.17 ± 15.75	40.38 ± 29.84	1.05 ± 0.01	2,552.42 ± 951
MEGAHIT	2(0)/12	2/23	4.71	68.01 ± 8.23%	324.83 ± 244.29	2254.09 ± 1901.7	1.92 ± 0.67	32,446.08 ± 35,925
PEHaplo	4(3)/12	4/12	5.70	52.72 ± 18.07%	54.0 ± 78.17	**13.04** ± **11.68**	1.05 ± 0.07	10,960.1 ± 6,602
tadpole	1(0)/12	1/12	3.15	24.47 ± 13.31%	39.92 ± 12.98	27.14 ± 50.67	**1.00** ± **0.00**	1,344.3 ± 3,292
ABySS	6(3)/12	6/12	6.41	64.88 ± 4.94%	20.92 ± 8.85	250.0 ± 85.39	1.05 ± 0.01	12,399.25 ± 5,157
Ray	4(4)/12	4/12	5.90	51.39 ± 2.27%	16.67 ± 12.53	67.54 ± 63.39	1.07 ± 0.06	26,154.75 ± 36,557
IDBA	0(0)/12	0/12	3.10	32.71 ± 9.52%	83.75 ± 13.24	104.46 ± 76.39	**1.03** ± **0.01**	154.67 ± 178
Vicuna	4(1)/12	4/12	4.18	47.26 ± 0.93%	36.33 ± 7.79	104.05 ± 71.31	**1.02** ± **0.01**	2,657.67 ± 704
IVA	4(3)/12	4/12	7.42	43.23 ± 16.2%	**11.92** ± **8.22**	121.61 ± 185.89	**1.02** ± **0.03**	**63,773.0 ± 49,449**
VirGenA	*6(5)/12*	6/12	7.63	47.25 ± 1.35%	*5.67* ± *2.48*	102.41 ± 107.01	*1.01* ± *0.00*	33,324.58 ± 30,049
SAVAGE	*9(5)/12*	9/17	4.61	*82.43* ± *15.69%*	283.17 ± 98.89	33.86 ± 28.37	1.46 ± 0.14	1,245.33 ± 349

Table S5: Genome fraction and NGA50 and their standard deviation for the high and low abundant strains in the HCMV in-vitro mixtures (two 1:10 and two 1:50 mixtures).

	metaSPAdes	MEGAHIT	Haploflow
Genome fraction (lower abundance)	19.77 ± 6.44%	30.00 ± 12.6%	**76.67** ± **12.24%**
Genome fraction (higher abundance)	**94.86** ± **0.57%**	89.97 ± 8.59%	91.62 ± 5.38%
NGA50 (lower abundance)	0 ± 0	0 ± 0	**9,625** ± **6,578**
NGA50 (higher abundance)	**149,712** ± **48,744**	45,481 ± 45,766	79,284 ± 50,679

Table S6: One cluster with 11 closely related phage strains from the simulated virome and the genome within-cluster similarities. Columns give the maximal or average ANI to all other sequences in the cluster.

GI number	Scientific name	Within-cluster similarity max	Within-cluster similarity avg
118725053	Staphylococcus phage phiNM3	98.10%	93.32%
119443652	Staphylococcus phage phiPVL108	98.31%	94.59%
157102936	Staphylococcus prophage tp310-1	99.12%	95.47%
157102938	Staphylococcus prophage tp310-3	98.74%	95.72%
239507361	Staphylococcus phage phiPVL-CN125	98.31%	94.59%
257136356	Staphylococcus phage P954	96.12%	92.18%
29028667	Staphylococcus prophage phi 13	98.74%	95.73%
30043925	Staphylococcus prophage phiN315	98.10%	92.98%
41189515	Staphylococcus phage 77	96.12%	93.13%
9635165	Staphylococcus phage PVL	99.12%	95.25%
9635677	Staphylococcus prophage phiPV83	96.59%	93.86%

Table S7: Genome fractions in % on different subsets of the simulated virome. Unique genomes refers to genomes for which no other genome with an ANI of >95% is in the data set, common strain genomes are ones for which at least one such genome is present. The coverage value was calculated by dividing the total number of base pairs in the reads belonging to the genome by its size.

	SPAdes	MEGAHIT	Haploflow
Common strains	55.58	48.88	**62.85**
Common strains, coverage > 8	74.58	64.99	**89.36**
Total	**72.2**	68.6	66.6
Total, coverage > 8	93.07	87.55	**94.52**

Table S8: Comparison of multiple strain infection labeling of samples by VATK[78], the predicted relative abundance of the low abundant strain(s) and the predicted abundance by Haploflow (relative and absolute) as well as the genome completeness (genome fraction, mapped against the first sample consensus genome) of strains Haploflow reconstructed (Supplementary methods). A ”-” denotes that no evidence of a second strain was found by either VATK (column 3) or Haploflow (column 4). Percentage values with a (*) denote problems in clustering, evident by a still high duplication ratio after clustering or the sum of genome fractions of two clusters summing up to ~1, indicating that underclustering or in the latter case overclustering took place. Three percentage values in the third column indicate that Haploflow predicted three strains being present.

Patient	Time points	Estimated low strain abundance (number of predicted strains)	Haploflow low strain abundance predictions (% and absolute value(s))	Genome fraction (portion of recovered genome) of strains vs. consensus sequence
RTR3	367 days	38.3% (2)	27.1% (43:16)	88.29% / 40.70%
	408 days	17.9% (2)	17.3% (167:35)	92.93% / 68.76%
RTR6	vitreous humour	9.4% (2)	12.7% (2576:373)	90.07% / 31.06%
	blood	17.3% (2)	18.4% (213:48)	99.07% / 92.73%
SCTR1	91 days	4.0% (2)	- (425)	99.69%
	126 days	22.9% (2)	21.2% (2429:653)	96.42% / 75.93%
	130 days	14.0% (2)	12.6% (313:45)	99.63% / 82.87%
	194 days	6.8% (2)	- (105)	99.78%
	224 days	37.8% (2)	26.8% (60:22)	92.73% / 79.36%
	231 days	25.4% (2)	23.6% (126:39)	92.09% / 37.63%
	244 days	- (1)	17.3% (81:17)	93.19%
	245 days	29.8% (2)	- (22)	92.86%
SCTR3	189 days	- % (1)	- (9)	98.74%
	272 days	20.5% (2)	24.3% (2781:893)	80.89% / 34.96%
	320 days	28.1% (2)	- (38)	98.46%

SCTR8	55 days	24.4% (2)	7.3% (178:14)	99.28% / 24.28%
	287 days	16.5% (2)	30.2%/8.3% (305:150:41)	93.86% / 58.5% / 52.33% (*)
SCTR11	88 days	15.6% (2)	16.3% (103:20)	99.07% / 85.76%
	192 days	11.6% (2)	11.2% (119:15)	91.75% / 78.20 %
SCTR17	21 days	34.7% (2)	22.1% (106:30)	94.81% / 30.96%
	28 days	30.6% (2)	17.6% (404:86)	98.39% / 17.04% (*)
	35 days	33.0% (2)	29.8%/9.5% (362:178:57)	99.46% / 28.08% / 22.61% (*)
	50 days	6.1% (2)	8.5% (4063:376)	97.26% / 94.18%
SCTR18	28 days	30.6% (3)	26.8% (101:37)	99.27% / 50.60% (*)
	35 days	30.3%/10.3% (3)	32.6%/10.2% (291:166:52)	80.79% / 81.88% / 48.85%
Summary strain detection		Total strains: 48	Recall: 91.7% (44/48)<br>Precision: 93.6% (44/47)	
